# The Induction of Labor in Nephritis

**Published:** 1895-02

**Authors:** William Brinton

**Affiliations:** Baltimore, Md.


### THE INDUCTION OF LABOR IN NEPHRITIS.*

BY WILLIAM BRINTON, M.D.,

BALTIMORE, MD.

I have been induced to bring the subject of the Induction of Labor in Nephritis to your notice by the reading of a paper on "The Significance of Albuminuric Retinitis in Pregnancy," written by Dr. R. L. Randolph of this city. Dr. Randolph reports five cases of albuminuric retinitis occurring in pregnant women whom he has seen during the past two years, in which cases he decided by ophthalmoscopic examination whether it was the proper treatment or not to induce labor for the purpose of saving the eyes and perhaps the life of the woman. In the cases related, not only were the eyes saved where labor was induced, but in the cases where he ad-

*Read before the Clinical Society of Maryland, December 7, 1894.

vised the continuation of pregnancy, the women escaped eclampsia. Judging from the first case reported by Dr. Randolph there must be some difference of opinion even among oculists as to when premature labor should be induced, for the report of this case which I shall now read will show that the first oculist consulted advised a different method of procedure from that recommended by Dr. Randolph.

CASE.—Mrs. M., thirty-one years old, three children living, and up to the fourth month of her third pregnancy had enjoyed good health. In the early part of the fifth month she began to have violent headaches, which could only be relieved by strong anodynes. They persisted for two weeks, when she noticed that her sight was growing dim. It continued to grow worse until she was practically blind in one eye and the sight in the other but little better. At this time an oculist was called in, pronouncing it albuminuric retinitis, and found the urine rich in albumin and some casts present. The induction of labor was advised, performed, and a dead child born. The woman had convulsions, but recovered with complete restoration of sight. One year later she again conceived, and in the fourth month was attacked with similar headaches. Fearing that her sight would again become bad, she consulted an oculist again, who advised that if she waited for normal labor she would lose her sight and probably her life. Dr. Kelly was sent for to induce labor, but referred the case first to me. I found the vision $\frac{20}{15}$ in both eyes, and a low grade of hyperopic astigmatism. I found absolutely nothing to denote progressive disease in the fundus. The question was whether or not to induce premature labor. There was a faint trace of albumin in the urine but no casts. I concluded that the evidence did not justify the operation. My advice was followed, and the patient sent home to give birth a few months later to a boy.

The conclusions of this interesting paper were as follows: 1st. Visual disturbances occurring in the first six months of pregnancy, and especially when associated with violent headaches, frequently mean albuminuric retinitis, and if this condition is found, to *save sight* pregnancy should be at once terminated. 2d. Visual disturbances showing themselves in the last seven weeks of pregnancy, while indicating the same retinal lesions, are of less gravid import in so far as sight is concerned, and unless they are very pronounced and associated with wide-spread ophthalmoscopic changes, should not in themselves call for the induction of labor. 3d. The occurrence of renal retinitis in one pregnancy does not mean that the woman will be likewise affected in a subsequent one. And even though headache be present and albumin found, so long as the fundi are free from signs of existing retinitis the question of sight will not be considered.

The very grave prognosis in cases of eclampsia occurring in the pregnant woman, the woman in labor, or the parturient, makes the question of nephritis a very interesting one to the obstetrician. Experience and statistics prove that women who have chronic nephritis, conceive and carry their children to full term without having convulsions. Indeed, it seems that if they do not abort they are less liable to eclampsia than women, who for the first time, develop kidney disease during pregnancy. Cases of nephritis occurring in the pregnant woman, whether chronic or acute in character, must make the physician in charge anxious about the outcome of the case, for the rates of mortality vary from twenty-five to forty per cent. for the mother and from fifty to seventy-five per cent. for the child when we have eclampsia occurring during pregnancy, or before the completion of pregnancy. The question comes to us for decision whether we shall follow conservative treatment, which at best will only ward off impending danger; or whether it is best to place the patient at once in a position of comparative safety by the induction of premature labor. Dr. Lusk says: "The weight of authority seems to me favorable to procrastination, the interruption of pregnancy being regarded as an extreme measure, justifiable only in case of utmost peril. But my own convictions are clear that so soon as grave cerebral symptoms develop, the period of folded hands has passed."

The four cases I shall report have come under my notice during the past eighteen months, and while in only two cases was premature labor induced, previous to convulsive movements, yet in the other two although only seen first when in convulsions, premature labor was induced, as they were not at full time.

CASE 1.—Mrs. R., mother of nine children and between seven and eight months advanced in her tenth pregnancy. Her physician had watched her closely for some weeks and made diagnosis of nephritis. He found albumin and casts in the urine, specific gravity 1010; eyesight very much impaired and rapidly growing worse; headaches violent for days and several times had had convulsive movements. At my first visit we decided upon premature labor and under strict antiseptic precaution. I introduced a bougie at four P. M. on Friday afternoon. At midnight of the next day she was delivered of a living child. During the time of the induction of labor she had to be kept under the influence of potassium bromide and chloral hydrate. For a week or two both mother and child did well, but finally all her symptoms grew worse, she became totally blind, went into coma, and died two months after the birth of her child.

CASE 2.—Mrs. A., forty years of age, pregnant for the ninth time and supposed to be eight months advanced. She was blind edematous, pulse rapid and urine full of albumin. There were very marked indications of beginning convulsions. Treatment had been: Infusion of digitalis, compound jalap powder, and chloral hydrate and bromide of potash. I introduced a bougie as in case 1. Hot vaginal douches were given, and some eleven hours after the mother was delivered of a living child. Some nine months after her physician writes me that the child died within a month, but that Mrs. A. recovered with good sight.

CASE 3.—A colored out-patient, with a history of eleven convulsions before my assistant saw her. An examination showed pregnancy of eight months. Child living, woman aged seventeen. She was removed to the hospital; chloroform, bromide of potash, and chloral hydrate given to control convulsions. Bougie was introduced, but later we had to dilate with the finger. Simpson's forceps were applied, and after great traction a dead child delivered. The mother never regained consciousness. Died four hours later, having had fifty or sixty convulsions.

CASE 4.—Mrs. V. C., in her first confinement. All during her pregnancy had been well; had been on the street the day previous and slept well that evening. In the morning while at breakfast she suddenly clapped her hands to her head, cried "I cannot see," and fell to the floor in violent convulsion. Within thirty minutes she had six more. Chloroform was given during convulsions and chloral every hour during the intervals, when the patient had intelligence enough to swallow when told to do so. With the assistance of Dr. Watson, dilatation was made by the finger; Simpson's forceps applied, and a living child delivered. The woman had in the next thirty-six hours about ten severe convulsions and was practically unconscious for forty-eight hours afterwards. Hypodermics of morphia of one-third of a grain were used, and we saw marked results for good after each dose. She gradually grew better, but complained of bad sight and violent headaches for nearly two weeks. She has done well ever since.

In the brief report of these cases I have only mentioned a few of the many methods of inducing premature labor, but in closing I wish to commend the method of dilating the cervix with the finger.

